# Chromatin-Remodeling Factor BRG1 Is a Negative Modulator of *L. donovani* in IFNγ Stimulated and Infected THP-1 Cells

**DOI:** 10.3389/fcimb.2022.860058

**Published:** 2022-04-01

**Authors:** Harsimran Kaur Brar, Gargi Roy, Akanksha Kanojia, Evanka Madan, Rentala Madhubala, Rohini Muthuswami

**Affiliations:** ^1^ Molecular Parasitology Laboratory, School of Life Sciences, Jawaharlal Nehru University, New Delhi, India; ^2^ Chromatin Remodeling Laboratory, School of Life Sciences, Jawaharlal Nehru University, New Delhi, India

**Keywords:** *L. donovani*, IFNγ responsive genes, STAT1α, BRG1, *CIITA*, *HDAC1*-siRNA

## Abstract

Intracellular pathogens manipulate the host cell for their own survival by contributing to modifications of host epigenome, and thus, altering expression of genes involved in the pathogenesis. Both ATP-dependent chromatin remodeling complex and histone modifications has been shown to be involved in the activation of IFNγ responsive genes. *Leishmania donovani* is an intracellular pathogen that causes visceral leishmaniasis. The strategies employed by *Leishmania donovani* to modulate the host epigenome in order to overcome the host defense for their persistence has been worked out in this study. We show that *L. donovani* negatively affects BRG1, a catalytic subunit of mammalian SWI/SNF chromatin remodeling complex, to alter IFNγ induced host responses. We observed that *L. donovani* infection downregulates BRG1 expression both at transcript and protein levels in cells stimulated with IFNγ. We also observed a significant decrease in IFNγ responsive gene, Class II transactivator (*CIITA*), as well as its downstream genes, *MHC-II* (*HLA-DR* and *HLA-DM)*. Also, the occupancy of BRG1 at *CIITA* promoters I and IV was disrupted. A reversal in *CIITA* expression and decreased parasite load was observed with *BRG1* overexpression, thus, suggesting BRG1 is a potential negative regulator for the survival of intracellular parasites in an early phase of infection. We also observed a decrease in H3 acetylation at the promoters of *CIITA*, post parasite infection. Silencing of *HDAC1*, resulted in increased *CIITA* expression, and further decreased parasite load. Taken together, we suggest that intracellular parasites in an early phase of infection negatively regulates BRG1 by using host HDAC1 for its survival inside the host.

## Introduction

Visceral leishmaniasis, a neglected tropical disease, is caused by the protozoan parasite *Leishmania donovani* (Herwaldt, 1999). *Leishmania* after infecting the host cells, modify the transcriptome and proteome content of their host cells, facilitating their survival and replication inside the macrophages (Croken et al., 2012; Cheeseman and Weitzman, 2015). Chromatin remodeling in host cells post *Leishmania* infection is yet another mechanism (Croken et al., 2012; Cheeseman and Weitzman, 2015).

Extensive chromatin remodeling and the assembly of the transcriptional machinery at gene promoters is a prerequisite for differential gene expression. Histone modifications, such as, acetylation, methylation, or phosphorylation at distinct residues are critical factors which controls gene expression (de Ruijter et al., 2003; Zupkovitz et al., 2006). These modifications dictate the accessibility of DNA to the required proteins for transcriptional activation or repression (de Ruijter et al., 2003; Zupkovitz et al., 2006). Histone acetyl transferases (HATs) and histone deacetylases (HDACs) are a set of enzymes that acetylates or deacetylates histones for activation or repression of transcription (de Ruijter et al., 2003; Zupkovitz et al., 2006; Kouzarides, 2007). Chromatin remodeling complexes are a second group of enzymes involved in chromatin regulation and disruption of histone-DNA contacts in an ATP-dependent manner (Chi, 2004). Brahma-related gene-1 (BRG1) is the central catalytic subunit of several chromatin-remodeling enzymatic complexes and plays a major role in differential gene expression through chromatin modulation (Trotter and Archer, 2008). The prototypic BAF (BRG/Brahma (BRM)-associated factor) complex is related to the yeast SWI/SNF complex and is vital for the expression of immune-related genes upon external stimuli (Chi, 2004). BRG1 has been shown to be a necessity for the Interferon-γ (IFNγ) induction of class II transactivator (*CIITA*) (Pattenden et al., 2002; Ni et al., 2005). *CIITA* is also reported to be the master regulator of major histocompatibility complex class II (MHC-II) cell surface receptor protein (Zika et al., 2003) which are required for presenting antigens to CD4^+^ T helper cells (Steimle et al., 1994). IFNγ, a cytokine produced by activated T lymphocytes, regulates immunologically responsive genes (Decker et al., 1991), *via* JAK/STAT pathway (Gotthardt and Sexl, 2016). BAF complexes containing BRG1 interact with histone-modifying enzymes to further regulate IFNγ responsive genes (Chi, 2004; Wright and Ting, 2006). *Leishmania* infection has been shown to affect the expression of essential macrophage activation signaling molecules (Forget et al., 2001; Marr et al., 2014; Singh et al., 2015; Fernandez-Figueroa et al., 2016). *L. donovani* has also been reported to repress JAK2/STAT1 signalling pathway and reduce STAT1 localization to the nucleus (Forget et al., 2005; Olivier et al., 2005). Expression levels of IFNγ induced MHC-II and inducible nitric oxide synthase (iNOS) were reported to be significantly reduced in *L. donovani* infected macrophages (Matte and Descoteaux, 2010). However, the effect of the parasite infection on host BRG1 has not yet been elucidated.

Taking into consideration that BRG1 is essential for IFNγ to regulate immunologically responsive genes, and the ability of *Leishmania* parasite to manipulate the host defense system, in this study, we have analysed the impact of *L. donovani* infection on host BRG1, and further affecting IFNγ responsiveness, using THP-1 cells as the model system (ThermoFisher Understanding Calculations for siRNA Data: % Remaining Gene Expression and % Knockdown). In our study, we have investigated the role of BRG1 in regulating the expression of IFNγ responsive gene, *CIITA* and *MHC-II*. We observed that *L. donovani* infection downregulates BRG1 which further decrease IFNγ responsive genes, *CIITA* and its downstream genes, *MHC-II* (*HLA-DR* and *HLA-DM)*, to disrupt the host immune system. A study by Zika et al., demonstrated that inhibition of histone deacetylases (HDACs) enhanced the expression of MHC class II cell surface receptor protein encoded by the human leukocyte antigen complex (HLA complex) (Zika et al., 2003). We in an earlier study showed that *L. donovani* regulates the host HDAC1 expression in their benefit to survive within the host (Roy et al., 2020). In the present study, we showed that silencing of *HDAC1* as well as overexpression of *BRG1* were able to recompense the *CIITA* levels followed by a significant decrease in intercellular parasite survivability.

## Materials and Methods

### Antibodies

BRG1 rabbit monoclonal antibody (Catalog No ab110641) was purchased from Abcam, UK. STAT1α rabbit polyclonal antibody (Catalog No SAB3500364-100UG) and β-actin mouse monoclonal antibody (Catalog No A1978-100UL) were purchased from Sigma-Aldrich, USA. Anti-mouse IgG, HRP-linked antibody (Catalog No 7076), and anti-rabbit IgG, HRP-linked antibody (Catalog No 7074S) was purchased from Cell Signaling Technology, USA. Alexa green 488-conjugated goat anti-rabbit IgG (Catalog No A-11070) was purchased from Thermo Fisher Scientific, USA.

### Parasite and Mammalian Cell Culture Conditions

*L. donovani* Bob (LdBob/strain/MHOM/SD/62/1SCL2D) (Goyard et al., 2003; Debrabant et al., 2004) acquired from Dr Stephen Beverly (Washington University, St. Louis, MO) and THP-1 cells (202 TIB; American Type Culture Collection, Rockville, MD) were cultured as described previously (Roy et al., 2020).

### Macrophage Infection

THP-1 cells (10^6^ cells/ml) were differentiated and infected as previously described (Roy et al., 2020). After infection, cells were washed with phosphate-saline buffer (PBS) and rested for 2 h, followed by 1 ng/ml IFNγ stimulation (catalogue no SRP3058-100UG, Sigma-Aldrich, USA) for 30 min. The cells were harvested at time points - 0, 3, 6, and 24 h. Infection was confirmed by Giemsa (Sigma-Aldrich, USA) and Propidium Iodide (PI) (Sigma-Aldrich, USA) staining.

### RNA Extraction and Quantitative Real-Time RT-PCR

Total RNA was isolated and used for quantitative real-time RT-PCR (qPCR) as described in (Roy et al., 2020). Expression of various genes was analyzed using their specific primers (**Table S1**). *RNU6A* was used as a housekeeping gene. The fold change values of different genes at 3, 6 and 24 h were normalized to the respective values at 0 h. The results were calculated by the 2^-ΔΔ^ *CT* method (Singh et al., 2015).

### Chromatin Immunoprecipitation

Recruitment of BRG1 and STAT1α proteins and H3 acetylation at the promoters of concerned genes was analyzed by ChIP assay using chromatin from infected and/or IFNγ stimulated THP-1 cells (10^6^ cells/ml). Further, qPCR was performed using promoter-specific primers (**Table S1**). The cells were harvested and processed for ChIP analysis as reported previously (Roy et al., 2020). BRG1, STAT1α and acetylated histone (Ac-H3) bound DNA was immunoprecipitated overnight at 4°C using BRG1 (1 µg/25 µg chromatin extract), STAT1α (2 µg/25 µg chromatin extract) and Ac-H3 (1 µg/25 µg chromatin extract) antibodies. To quantify the DNA isolated by ChIP, qPCR was performed using primers spanning -288 to -99 of *CIITA* promoter I and -158 to +21 of *CIITA* promoter IV (**Table S1**). The change in gene expression for relative quantification was calculated by 2^^−ΔΔCT^ method (Singh et al., 2015). For calculating relative enrichment of each DNA fragment, fold change difference of the C_T_ values concerning the no antibody control and 0 h chromatin extract control was used.

### Immunoblotting

To study STAT1α and BRG1 protein expression, infected and/or stimulated THP-1 cells were lysed in urea buffer (90% 8.8 M urea, 2% 5 M NaH_2_PO_4_, 8% 1 M Tris-Cl pH 8) at 4°C. 80 μg of total protein was separated on 8% SDS-PAGE by electrophoresis (Bio-Rad Laboratories, USA). Immunoblotting was performed as described before (Roy et al., 2020) using STAT1α (1:500), BRG1 (1:1000) and β-actin (1:2000) specific antibodies. The membrane was then washed with Tris-buffered saline (TBS) and incubated with horseradish-peroxidase (HRP)-conjugated anti-mouse (1:3000) or anti-rabbit (1:1000) IgG antibody. The complexes were visualized by ECL chemiluminescence. Protein expression was normalized with the corresponding β-actin and quantitated by densitometry using ImageJ software.

### Immunofluorescence Microscopy

THP-1 cells were differentiated on coverslips followed by infection and stimulation as mentioned above. After 6 h, the cells were fixed and permeabilized with 0.5% Triton X-100 and then blocked in 2% BSA. The cells were probed with STAT1α antibody (1:50) followed by incubation with Alexa green 488-conjugated goat anti-rabbit IgG (1:200). DAPI (1 μg/ml) was used to stain the host nuclei and parasite kinetoplastid DNA. All antibody incubations were followed by washes with 0.2% Triton X-100. The images were then visualized under a confocal laser scanning microscope (Olympus FluoViewTM FV1000) at 488 nm wavelength.

### *BRG1* Overexpression

THP-1 cells (10^6^ cells/ml) were differentiated, followed by parasite infection and IFNγ stimulation as mentioned above. Subsequently, the cells were transiently transfected with 1.5 μg of plasmid overexpressing *BRG1* (Patne et al., 2017) for 48 h, using lipofectamine 3000 (Catalog No L3000015, Thermo Fisher Scientific, USA). The transfection reagents were mixed according to the manufacturer’s protocol. The mRNA expression levels of concerned genes were examined by qPCR. THP-1 cells transfected with vector (pcDNA3.1 LAP-Zeo) alone was used as a negative control.

### Small Interference RNA Transfection

The PMA treated differentiated THP-1 cells (10^5^ cells/ml) were transiently transfected with 600 pmole (Garcia-Garcia et al., 2009) of siGENOME Human *HDAC1* (3065) siRNA – SMARTpool (Dharmacon, USA) using lipofectamine 3000. The cells were incubated with siRNA for 24 h to allow gene silencing. Subsequently, the cells were washed for infection and stimulated as described earlier. THP-1 cells were harvested after 6 h and mRNA expression of *HDAC1* and *CIITA* genes was analyzed by qPCR. ON-TARGET plus Control Pool (Dharmacon, USA) was used as a negative control. The basal level of *HDAC1* and *CIITA* in uninfected Sc-siRNA transfected cells respectively were used for data normalization and were taken as 1.0. The transfection efficiency was calculated (ThermoFisher) to be >50%, as has also been reported by the manufacturer (Dharmacon, USA).

### Intracellular Parasite Load

THP-1 cells (10^5^ cells/ml) were infected and stimulated with IFNγ as mentioned earlier. After 6 h, for visualization of intracellular parasites, Giemsa staining was performed, and the parasite load was calculated (Manhas et al., 2014; Roy et al., 2020).

### Statistical Methods

GraphPad Prism (version 5.0) software (GraphPad Software, Inc.) was used for plotting data. Statistical analysis was measured using ANOVA. *P ≤* 0.05 was considered significant [* (*P ≤* 0.01 to 0.05), ** (*P*≤ 0.001), *** (*P*≤ 0.0001), **** (*P*≤ 0.0001), ns (*P*≥ 0.05)]. Error bars used in the figures specify standard error of the mean (Cheeseman and Weitzman).

## Results

### BRG1 Expression Is Downregulated in IFNγ Stimulated and Infected THP-1 Cells

In earlier reports and in our earlier studies, THP-1 cells were incubated with parasites for 3 h (Roy et al., 2020), followed by 2 h of resting period (Forget et al., 2005) prior to IFNγ stimulation (Forget et al., 2005, Lang et al., 2012). Significant IFNγ response has been demonstrated, within 30 min to 6 h of IFNγ stimulation (Blanchette et al., 1999; Forget et al., 2005; Forget et al., 2006; Matte and Descoteaux, 2010, Lang et al., 2012, Singh et al., 2015; Roy et al., 2020). Based on these studies, we designed all our experiments to study the effect of *Leishmania* infection in the host cells at an initial stage of infection. The impact of IFNγ on parasite load within the infected macrophages was analyzed by visually counting the intracellular amastigotes after Giemsa staining (**Figure S3**). A comparable parasitemia count (6h: 8/macrophage and 6/macrophage; 24 h: 13/macrophage and 11/macrophage) between non-stimulated and stimulated cells at both 6 and 24 h was observed showing that IFNγ stimulation has no detrimental effect on the intracellular parasite load.

**Figure 1:**
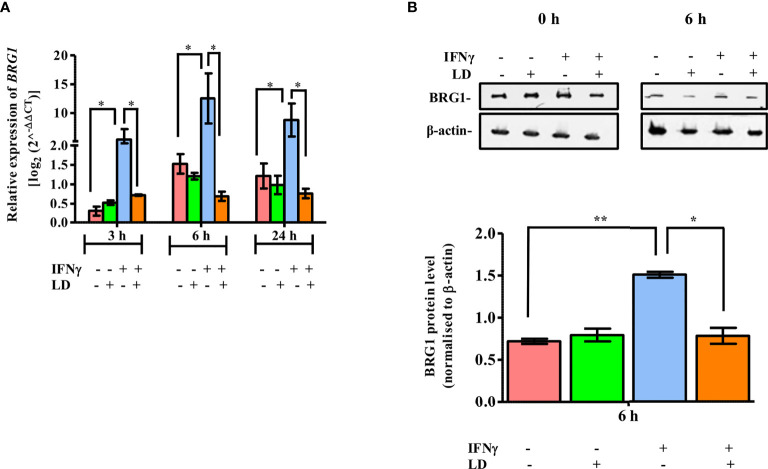
Expression of BRG1 is downregulated in IFNγ stimulated and infected THP-1 cells. Uninfected and infected THP-1 cells were stimulated or not with IFNγ for 30 min. **(A)** Cells were harvested at 0, 3, 6 and 24 h post-stimulation for the analysis of *BRG1* mRNA expression in host cells by qPCR. **(B)** Cells harvested at 0 and 6 h post-stimulation were lysed in urea buffer. After separation on SDS-PAGE, BRG1 protein expression was analyzed by immunoblotting. BRG1 protein levels were normalized with corresponding β-actin levels and quantitated by densitometry using ImageJ software. The results are mean ± SEM of three independent experiments. For calculating statistical significance, ANOVA was used. P-value for significance: *P ≤ 0.01 to 0.05, **P ≤ 0.001. Pink color represents: - IFNγ - *L. donovani*; green color represents: - IFNγ + *L. donovani*; blue represents: + IFNγ - *L. donovani*; orange color represents: + IFNγ + *L. donovani*.

Hence, we first investigated the expression of BRG1 in response to IFNγ stimulation at 3, 6 and 24 h, post *Leishmania donovani* infection, by qPCR. The data showed that there was no significant alteration in the expression of *BRG1* between infected and uninfected THP-1 cells under unstimulated condition (**Figure 1A**). However, the mRNA levels of *BRG1* were significantly upregulated at 3 h (~ 18 fold, *P* = 0.01), 6 h (~ 8.3 fold, *P* = 0.019) and 24 h (~ 7.3 fold, *P* = 0.014) in uninfected and stimulated cells as compared to the resting macrophages. This is similar to the upregulation of *STAT1α* observed in the previous studies (Ray et al., 2000; Forget et al., 2005) and in **Figure S1A**. On infection and stimulation with IFNγ, the expression of *BRG1* decreased significantly at 3 h (~ 86.6%, *P* = 0.016), 6 h (~ 94.4%, *P* = 0.012) and 24 h (~ 91%, *P* = 0.01) in comparison to the uninfected, stimulated cells (**Figure 1A**).

Next, we evaluated the expression of BRG1 protein in response to IFNγ stimulation in THP-1 cells. Cells were harvested at 6 h since at this time point maximal increase in *BRG1* expression was observed in stimulated uninfected cells as compared to the resting macrophages (**Figure 1A**). BRG1 protein levels were checked by western blot and analysed by densitometry. As with the expression of mRNA levels, there was no significant change in the protein expression between uninfected and infected cells under unstimulated condition (**Figure 1B**). On stimulation with IFNγ, BRG1 expression was ~ 2 fold (*P* = 0.004) higher in uninfected cells as compared to the resting macrophages (**Figure 1B**). BRG1 protein expression decreased significantly (~ 48.3%, *P* = 0.0195) on infection as compared to the uninfected, stimulated cells (**Figure 1B**).

It is well established that normal macrophage functioning with *Leishmania* infection is disrupted at early time points (Blanchette et al., 1999; Forget et al., 2006; Singh et al., 2015; Roy et al., 2020). To confirm that the results obtained in our study are because of live parasite infection, we have used uninfected cells (Matte and Descoteaux, 2010) and 0 h time points as controls. These control macrophages were incubated with or without parasites for 3 h, followed by washes and 2 h resting period and then 30 min IFNγ stimulation and then harvested to count as 0 h infected or uninfected. We have performed Giemsa and propidium iodide staining to confirm internalization of parasites inside the macrophages by 3 h of incubation (data not shown). We have also performed a similar infection experiment with heat-killed *L. donovani* (HKLD) instead of live parasites. qPCR data clearly demonstrated that HKLD infection did not have any effect on the regulation of *BRG1* expression (**Figure S4A**), thus, suggesting that live parasite infection plays a potential role for the downregulation of the *BRG1* expression, even after IFNγ stimulation. Similar results, showing no regulatory effect of HKLD on *STAT1α* expression were observed (**Figure S4B**).

### BRG1 Occupancy Is Reduced on *CIITA* Promoters Upon *L. donovani* Infection

**Figure 2:**
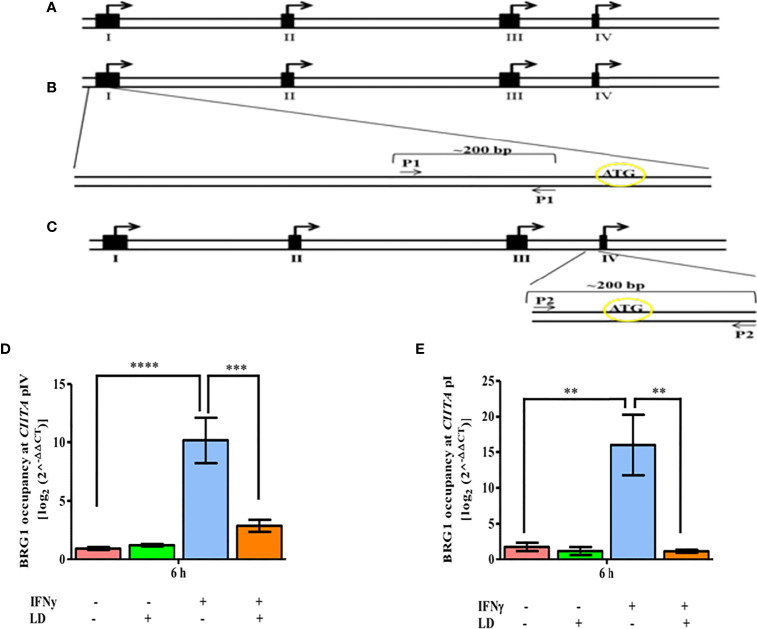
*L. donovani* infection reduces the BRG1 occupancy at CIITA in IFNγ stimulated cells. **(A)** Schematic diagram of 5’-flanking regions of the human *CIITA* gene. The solid black boxes denote the different first exons of *CIITA* promoters while the small open boxes represent introns. Arrows represent the major initiation sites. **(B)** For analyzing the occupancy of relevant factors on promoter I of human *CIITA* gene, primer spanning -288 to -99 of *CIITA* promoter I was designed (P1). **(C)** For promoter IV, primer spanning -158 to +21 of *CIITA* promoter IV was designed (P2). THP-1 cells were infected with *L. donovani* for 3 h at an MOI of 20:1, following which they were rested for 2 h. Cells were further stimulated with IFNγ for 30 mins or left unstimulated and harvested at 0 and 6 h post-stimulation. The occupancy of BRG1 at *CIITA* promoter IV **(D)** and promoter I **(E)** was analysed by ChIP using an antibody against BRG1. Immunoprecipitated DNA fragments were amplified by qPCR using promoter-specific primers. The 2^-ΔΔ^ *CT* method was used to measure the BRG1 occupancy levels. No antibody and 0 h chromatin extract were used as controls. The results are mean ± SEM of three independent experiments. ANOVA was used for measuring statistical significance. P-value for significance: **P ≤ 0.001, ***P ≤ 0.0001, ****P ≤ 0.0001. Pink color represents: - IFNγ - *L. donovani*; green color represents: - IFNγ + *L. donovani*; blue represents: + IFNγ - *L. donovani*; orange color represents: + IFNγ + *L. donovani*.

In mammals, *CIITA* expression is controlled by multiple promoters and is activated in a selective manner (Muhlethaler-Mottet et al., 1997) (**Figure 1A**). *CIITA* promoters I and III regulates the expression of *MHC-II* in dendritic cells and B cells, respectively (Muhlethaler-Mottet et al., 1997). Promoter IV has been demonstrated to mediate IFNγ inducible expression of *MHC-II* genes (Muhlethaler-Mottet et al., 1997). IFNγ induction is also reported to increase not only type IV but also type I *CIITA* mRNA levels in bone marrow-derived macrophage (BMM) cells (Pai et al., 2002). Since *CIITA* promoter IV (pIV) and promoter I (pI) both mediate IFNγ inducible gene expression, we hypothesized that the occupancy of BRG1 would be reduced on these promoters leading to the decreased expression of *CIITA*. Therefore, we checked the occupancy of BRG1 on *CIITA* pIV and also for the first time on pI (**Figure 2**).

ChIP experiments showed that the occupancy of BRG1 on pIV and pI was not significantly altered between infected and uninfected cells under unstimulated conditions (**Figures 2D, E**). On stimulation, the occupancy of BRG1 increased on both *CIITA* pIV (~ 10 fold, *P* = 0.0001) and pI (~ 9 fold, *P* = 0.003) in uninfected cells as compared to the resting macrophages. Further, the occupancy of BRG1 decreased significantly on both pIV (~ 72.5%, *P* = 0.0016) and pI (~ 92.7%, *P* = 0.002) in infected, stimulated cells in comparison to uninfected, stimulated cells (**Figures 2D, E**). Thus, the binding pattern of BRG1 to the *CIITA* promoters is in concordance with the *CIITA* expression pattern (**Figure S2A**). The occupancy of BRG1 on the promoter of DNA topoisomerase 1 (*TOP1*) was also checked, which served as a negative control. No occupancy of BRG1 at *TOP1* promoter region was observed (**Figure S5B**), suggesting a specific occupancy of BRG1 at *CIITA* promoters.

Thus, we conclude that in infected and stimulated THP-1 cells, disruption of the occupancy of BRG1 at both the *CIITA* promoters has a potential role in decreased *CIITA* transcription

### STAT1α Occupancy Is Reduced on *CIITA* Promoters Upon *L. donovani* Infection

Earlier studies have reported that JAK2/STAT1 signalling in host macrophages is negatively regulated on *Leishmania* infection (Nandan and Reiner, 1995; Blanchette et al., 1999; Bhardwaj et al., 2005). It is very well established that STAT1α, and its downstream IFNγ responsive genes *CIITA* and *MHC-II (HLA-DR and HLA-DM)* are downregulated in IFNγ stimulated host cells post *Leishmania* infection (Ray et al., 2000; Forget et al., 2005; Matte and Descoteaux, 2010; Singh et al., 2019). In **Figure S1A, B**, we also demonstrated that parasitic infection had an inhibitory effect on the STAT1α expression, both at the mRNA (at time points, 3, 6 and 24 h, post infection) and protein level at 6 h post infection, even in the presence of IFNγ. Further, the localization of STAT1α to the nucleus was also suppressed in infected and stimulated THP-1 cells as observed by immunofluorescence microscopy (**Figure S1C**). The anti-STAT1α fluorescence intensity in the host cell nuclei also showed a similar pattern (**Figure S1D**). We also observed downregulation of *CIITA* and *MHC-II* in parasite infected THP-1 cells (**Figure S2**).

The transcription factor, STAT1α, has been shown to be important for the expression of IFNγ responsive genes like *MHC-II* (Darnell et al., 1994). CIITA has been reported to play an important role in the expression of *MHC-II* in a STAT1-dependent manner (Muhlethaler-Mottet et al., 1997). (Pai et al., 2002). Further, in our previous results, we observed reduced STAT1α (**Figure S1**), CIITA (**Figure S2**) and BRG1 expression (**Figure 1**) in infected, IFNγ stimulated cells. Studies have shown that STAT1α binding to promoters of IFNγ responsive genes such as *CIITA* and *GBP1*, is dependent on BRG1 (Ni et al., 2005). Therefore, we hypothesized that the occupancy of STAT1α at the *CIITA* promoters in infected and stimulated THP-1 cells would also be reduced, leading to downregulation of *CIITA* expression.

**Figure 3:**
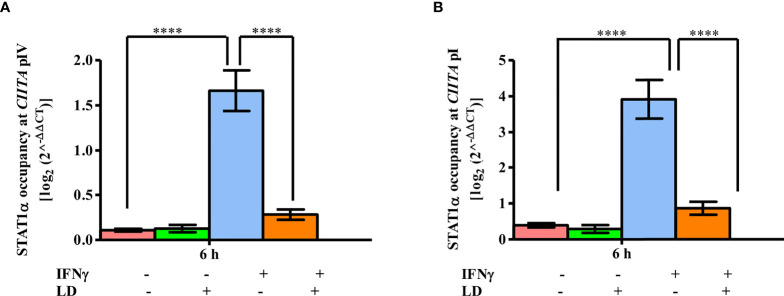
STAT1α binding to *CIITA* promoters is reduced on *L. donovani* infection. *L. donovani* infected and uninfected THP-1 cells (10^6^ cells/ml) were stimulated with IFNγ for 30 min or were left unstimulated. Cells were harvested at 0 and 6 h post-stimulation. ChIP assay was performed to study the occupancy of STAT1α on *CIITA* promoter IV **(A)** and promoter I **(B)** using the antibody against STAT1α. The immunoprecipitated DNA fragments were quantified by qPCR using promoter-specific primers. The STAT1α occupancy levels were expressed using the 2^-ΔΔ^ *CT* method. For calculating relative abundance, fold change difference of the C_T_ values of each sample concerning concerning no antibody and 0 h control was used. The results are mean ± SEM of three independent experiments. ANOVA was used for measuring statistical significance. P-value for significance: ****P ≤ 0.0001. Pink color represents: - IFNγ - *L. donovani*; green color represents: - IFNγ + *L. donovani*; blue represents: + IFNγ - *L. donovani*; orange color represents: + IFNγ + *L. donovani*.

Thus, the occupancy of STAT1α between infected and uninfected cells was checked at both *CIITA* pIV and pI (**Figure 3**). No change in occupancy levels was observed in unstimulated cells. However, on stimulation, the occupancy of STAT1α was dramatically increased on *CIITA* pIV (~ 15 fold, *P* = 0.00001) and pI (~ 10 fold, *P* = 0.00001) in uninfected cells when compared to the resting macrophages. On infection, the occupancy of STAT1α decreased significantly at both the promoters (pIV: ~ 83%, *P* = 0.00004; pI: ~ 77%; *P* = 0.0001) as compared to the uninfected, stimulated cells. No binding of STAT1α at *TOP1* promoter region was detected (**Figure S5A**), thus, suggesting a specific occupancy of STAT1α on *CIITA* promoters, Therefore, based on these results, we conclude that the decreased occupancy of STAT1α on *CIITA* promoters leads to decreased *CIITA* transcription.

Taken together, we conclude that in infected and stimulated THP-1 cells, disruption of the occupancy of both STAT1α (**Figure 3**), and BRG1 (**Figure 2**), at *CIITA* promoters leads to decreased *CIITA* transcription.

### BRG1 Is a Negative Regulator of Parasite Survival

**Figure 4:**
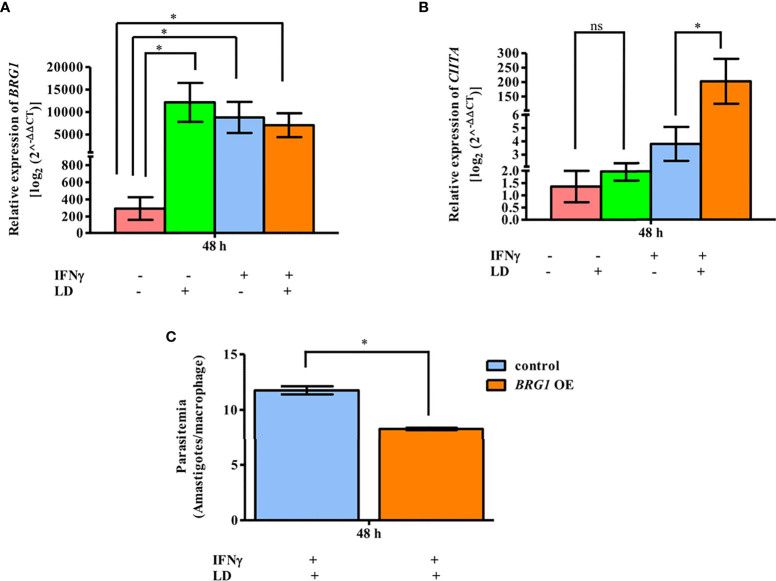
BRG1 negatively regulates parasite survival. After 2 h of rest, infected and uninfected THP-1 cells (10^6^ cells/ml) were either given stimulation with IFNγ for 30 min or left unstimulated. Cells were transfected with plasmid overexpressing *BRG1* (1.5 µg), using lipofectamine 3000. After 48 h of transfection, THP-1 cells were harvested for total RNA isolation. The mRNA expression of *BRG1* **(A)** and *CIITA* **(B)** were quantified by qPCR. Vector (pcDNA3.1 LAP-Zeo) alone transfected THP-1 cells were used as a negative control. The mRNA levels were measured using the 2^-ΔΔ^ *CT* method. RNU6A was used as a housekeeping gene. **(C)** THP-1 cells (10^5^ cells/ml) were infected or not with *L. donovani* (MOI, 20:1) for 3 h, rested for 2 h and then stimulated with IFNγ for 30 min. Cells were transfected with plasmid overexpressing *BRG1*. After 48 h, cells were fixed and stained with Giemsa stain for visual counting of intracellular parasite load (amastigotes). The graph in **(C)** represents the per cent amastigote viability. The results represent mean ± SEM of three independent experiments. For measuring statistical significance, ANOVA was used. P-value for significance: ns *P* > 0.05), *P ≤ 0.01 to 0.05, **P ≤ 0.001. Pink color represents: - IFNγ - *L. donovani*; green color represents: - IFNγ + *L. donovani*; blue represents: + IFNγ - *L. donovani*; orange color represents: + IFNγ + *L. donovani*.

BRG1, a core subunit of the BAF complex, is required for the expression of certain IFNγ responsive genes (Chi, 2004). As observed that reduced BRG1 occupancy is correlated with reduced *CIITA* expression (**Figure 2**), we hypothesized that the overexpression of BRG1 would lead to increased *CIITA* mRNA levels in parasite infected cells, and thus, negatively impact the survival of the parasite within the macrophages. To test this hypothesis, THP-1 cells were transiently transfected with the plasmid overexpressing *BRG1* and the transcript levels of the genes of interest were analyzed by qPCR. As expected, *BRG1* expression increased in all the conditions in the cells transfected with plasmid overexpressing *BRG1* as compared to vector alone (**Figure 4A**). The expression of *CIITA* in unstimulated cells was comparable between uninfected and infected cells (**Figure 4B**). However, in stimulated cells, the *CIITA* mRNA was upregulated (~ 53.4 fold, *P* = 0.0235) significantly in infected cells as compared to the uninfected one (**Figure 4B**).

To determine the intracellular parasite load in these conditions, infected and stimulated cells were used for visually counting the amastigotes within the THP-1 cells after Giemsa staining (**Figure 4C**). A significant decrease in the parasitemia (~ 29.4%, *P* = 0.012) was observed in infected and stimulated cells overexpressing *BRG1*, compared to infected and stimulated cells overexpressing only vector (**Figure 4C**). The parasite load in control samples, unstimulated and infected cells, was unchanged between cells overexpressing *BRG1* and vector alone (**Figure S6**). Taken together, this data demonstrates that BRG1 has a potential negative effect on parasite survival.

### *L. donovani* Impairs H3 Acetylation to Further Downregulate the Expression of IFNγ Responsive Gene *CIITA*

**Figure 5:**
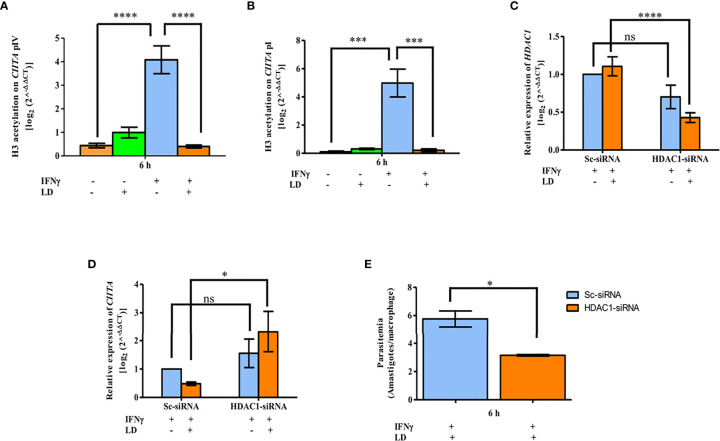
*L. donovani* impairs H3 acetylation, with the help of HDAC1, to downregulate *CIITA* expression. Uninfected and infected THP-1 cells were stimulated or not with IFNγ. Cells were harvested after 6 h of stimulation. ChIP assay was performed to analyse the H3 acetylation pattern at*CIITA* promoter IV **(A)** and promoter I **(B)**. Chromatin was pulled down using anti-acetylated lysine antibody. Immunoprecipitated DNA was quantified by qPCR using primers against the *CIITA* promoters. No antibody and 0 h chromatin extract were used as controls. **(B)** Differentiated THP-1 cells were transfected with 600 pmole of *HDAC1-* siRNA using lipofectamine 3000 for 24 h. Cells transfected with scrambled-siRNA was used as a negative control. Cells were washed and either infected with *L. donovani* for 3 h or not, followed by stimulation with IFNγ for 30 min. After 6 h, cells were harvested and total RNA isolated followed by qPCR to study the effect of HDAC1 silencing on *CIITA* expression. Expression of *HDAC1* **(C)** and *CIITA* **(D)** as enumerated by qPCR. *RNU6A* was used as a housekeeping gene. For data normalization, respective values of *HDAC1* and *CIITA* in uninfected-Sc-siRNA were used and taken as 1.0. The H3 acetylation levels and mRNA expression levels of *HDAC1* and *CIITA* were expressed using the 2^-ΔΔ^ *CT* method. **(E)** For observing the influence of HDAC1 silencing on the intracellular parasite load, cells were fixed with methanol after 6 h of stimulation and stained with PI stain for visually counting the parasites. The graph demonstrates the per cent amastigote viability in *HDAC1*-siRNA transfected cells compared to Sc-siRNA. The results are mean ± SEM of three independent experiments. Significance was calculated using ANOVA. P-value for significance: ns *P* > 0.05, *P ≤ 0.01 to 0.05, **P ≤ 0.001, ***P ≤ 0.0001, ****P ≤ 0.0001. Pink color represents: - IFNγ - *L. donovani*; green color represents: - IFNγ + *L. donovani*; blue represents: + IFNγ - *L. donovani*; orange color represents: + IFNγ + *L. donovani*.

Enhanced transcription of genes like *MHC-II* and *GBP2* in response to IFNγ is dependent on the acetylation of histones like H3 and H4 (Zika et al., 2003; Ramsauer et al., 2007). BRG1 is known to bind to acetylated histones (Shen et al., 2007). Therefore, we hypothesized that the global levels of H3 acetylation would decrease on the *CIITA* promoters on infection under stimulated condition. THP-1 cells were infected and stimulated as mentioned before. Cells were harvested at 6 h and analyzed for global H3 acetylation levels by ChIP assay. In unstimulated cells, the acetylation levels were low in both uninfected and infected cells (**Figure 5A**). On stimulation, a significant increase in the total H3 acetylation at CIITA pIV (~ 9 fold, *P* = 0.00003) and pI (~ 49 fold, *P* = 0.0002) in uninfected cells was observed as compared to the resting macrophages (**Figures 5A, B**). Further, as expected, in stimulated cells the acetylation levels decreased significantly on both the promoters in infected cells as compared to uninfected cells (pIV: 90%, *P* = 0.00002; pI: 95.7%, *P* = 0.0002) (**Figures 5A, B**). Thus, these results confirm our hypothesis.

In our earlier study, we have reported significant upregulation of HDAC1 in THP-1 cells on *Leishmania* infection (Roy et al., 2020). Therefore, to investigate the role of HDAC1 in the reduced H3 acetylation levels, THP-1 cells were transfected either with *HDAC1*-siRNA or scrambled-siRNA (Sc-siRNA). In THP-1 cells transfected with Sc-siRNA, comparable levels of *HDAC1* were observed in uninfected and infected conditions showing that Sc-siRNA has no inhibitory effect on host *HDAC1* expression. When THP-1 cells were transfected with *HDAC1*-siRNA, a decrease in *HDAC1* expression was observed in both infected (~ 62%, *P* = 0.00009) and uninfected (~ 30%, *P* = 0.0674) cells. This data confirms a specific silencing effect of *HDAC1*-siRNA on the expression of host *HDAC1* (**Figure 5C**).

The expression of *CIITA* in cells transfected with Sc-siRNA showed basal levels (**Figure 5D**). In cells transfected with *HDAC1*-siRNA, the *CIITA* levels were upregulated significantly in both infected (~ 4.8 fold, *P* = 0.0177) and uninfected (~ 1.5 fold, *P* = 0.28) cells. Thus, suggesting that downregulation of *HDAC1* is beneficial for the expression of *CIITA*.

To determine the role of *HDAC1*-siRNA on parasite load, visual counting of intracellular amastigotes was done after Giemsa staining (**Figure 5E**). In infected and stimulated cells, the parasite load (~ 45.25%, *P* = 0.04) was significantly downregulated in cells transfected with *HDAC1*-siRNA as compared to Sc-siRNA. This data demonstrates the positive effect of host HDAC1 on parasite survival.

The H3 acetylation pattern at both the *CIITA* promoters (**Figures 5A, B**) is in concordance with the mRNA expression pattern of *CIITA* in similar conditions (**Figure S2A**). Further in **Figures 5C** **–E**, a significant increase in *CIITA* expression whereas a decrease in the parasite viability upon *HDAC1* silencing was observed in infected and stimulated cells. Taken together, these results suggest that parasite infection leads to reduced host H3 acetylation at *CIITA* pI and pIV with the help of HDAC1, leading to decreased *CIITA* transcription.

## Discussion

*Leishmania* is known for its ability to alter macrophage signaling that is detrimental to its survival (Kwan et al., 1992; Proudfoot et al., 1996). Some of these signaling pathways are induced by cytokines such as IFNγ (Kwan et al., 1992; Proudfoot et al., 1996). Previous studies revealed that the parasite targets JAK2/STAT1α signaling cascade to reduce IFNγ inducible macrophage gene expressions (Nandan and Reiner, 1995; Blanchette et al., 1999; Martiny et al., 1999; Nandan et al., 1999). To investigate the role of epigenetic factors in this alteration of the gene expressions on *Leishmania* infection, we have used THP-1 cells as a model system. Here, we have shown that the crosstalk between STAT1α, BRG1, histone acetylation, and HDAC1 is responsible for the repression of *CIITA* and *MHC-II* genes on *Leishmania* infection. In accordance with the earlier study, stimulation of THP-1 cells with IFNγ upregulated *STAT1α * expression (Ray et al., 2000; Forget et al., 2005). *BRG1* mRNA expression was also increased by IFNγ, which was mimicked at the protein level too. Concomitantly, the expression of *CIITA* and therefore, *HLA-DM* and *HLA-DR* was also upregulated, as previously reported (Matte and Descoteaux, 2010; Singh et al., 2019). On parasite infection, downregulation of *BRG1* and *STAT1α* expression was observed leading to repression of *CIITA*.

ChIP studies showed that the occupancy of BRG1 and STAT1α on *CIITA* pI and pIV increased on stimulation with IFNγ which significantly decreased with *L. donovani* infection. This correlates with the transcript levels of *CIITA* and further *HLA-DM* and *HLA-DR*. Earlier studies have demonstrated the occupancy of STAT1 and BRG1 at various distal enhancers (Ni et al., 2008), as well as at pIV of *CIITA* (Ni et al., 2005). However, the occupancy of STAT1α and BRG1 on *CIITA* pI was a novel finding in our study. To get a better and clearer picture of the regulation of *CIITA* pI, epigenetic changes occurring at the promoter could be further examined in detail.

*Leishmania* infection also downregulated BRG1 expression, which led us to hypothesize that the protein might be a negative regulator for *Leishmania* infection. Indeed, overexpression of BRG1 led to increased *CIITA* expression and reduced parasite load within the host, validating our hypothesis. Therefore, we conclude that parasite infection leads to reduced BRG1 expression, further downregulating *CIITA* and its downstream genes. This downregulation of CIITA aids in the survival of the parasite inside host macrophages. Overexpression of BRG1 disrupts this approach of the parasite, thus, limiting its survival within the host cells.

As per previous studies (Forget et al., 2005; Olivier et al., 2005), we also show that STAT1α translocation to the nucleus in IFNγ stimulated cells was hindered on *Leishmania* infection. Thus, the decreased occupancy of STAT1α on *CIITA* promoters could be due to two reasons, i.e. decreased expression of *STAT1α* as well as retention of the protein in the cytoplasm.

The other major epigenetic player in the regulation of gene expression is histone acetylation (Galan and Cossart, 2005; Cheeseman and Weitzman, 2015). Histone acetylation is strongly associated with transcriptional activation while deacetylation results in transcriptional repression (de Ruijter et al., 2003; Zupkovitz et al., 2006). Previously, we have demonstrated that host HDAC1 is upregulated on *Leishmania* infection and further inhibition of host HDAC1 was detrimental for the parasite survival within the hosts (Roy et al., 2020). In our present study, we found that the global H3 acetylation decreases on the *CIITA* promoters on *Leishmania* infection in THP-1 cells stimulated with IFNγ. Further, silencing of *HDAC1* resulted in the reversion of *CIITA* expression indicating that histone H3 deacetylation is an important player in establishing parasite within the host cell at an early stage of infection.

**Figure 6:**
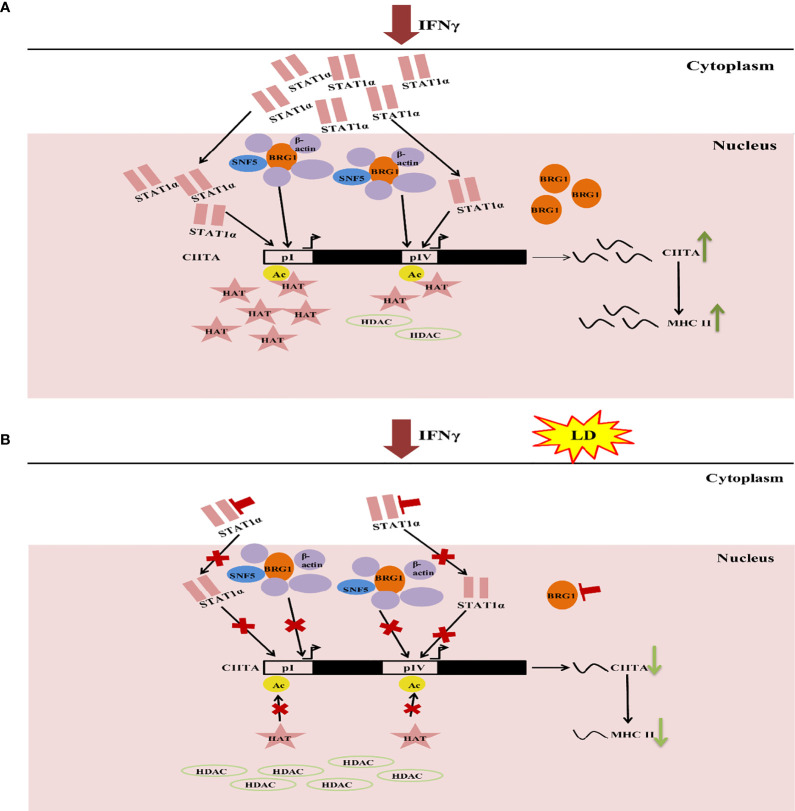
Model explaining the epigenetic changes that occur in macrophage on *Leishmania* infection. **(A)** On addition of IFNγ to THP-1 cells, the JAK-STAT pathway is activated. This leads to upregulation of STAT1α expression. Following this, STAT1α translocate into the nucleus and binds to the *CIITA* pI and pIV. Concomitantly, H3 acetylation increases on these promoters leading to increased occupancy of BRG1. Together, these three factors lead to increased *CIITA* expression. This, in turn, aiding in the transcription of *MHC-II* genes (*HLA-DR, HLA-DM*). **(B)** Infection with *L. donovani* (Herwaldt), increases histone deacetylase (HDAC) levels thereby supercoiling of the chromatin and thus, preventing expression of STAT1α and BRG1. This leads to a downregulation in the occupancy of these factors on the *CIITA* pI and pIV. H3 acetylation levels also decrease at the *CIITA* promoters (I and IV) resulting in downregulation of *CIITA* expression. This, in turn, reduces the expression of the downstream IFNγ responsive genes such as *CIITA, MHC-II* genes *(HLA-DR, HLA-DM*), thus, facilitating the establishment of parasite infection in the host cells.

These studies provide a glimpse into the role of the host epigenetics in *Leishmania* infection providing a testable model (**Figure 6**). IFNγ activates the JAK/STAT pathway (Gotthardt and Sexl, 2016; Lee and Ashkar, 2018) resulting in increased STAT1α and BRG1 expression. Thereby, translocation of STAT1α into the nucleus also increased, leading to its higher occupancy on *CIITA* promoters. Simultaneously, there is an increase in H3 acetylation levels as well as BRG1 occupancy on *CIITA* promoters leading to its increased transcription. CIITA, in turn, activates the expression of *MHC-II* genes (*HLA-DR and HLA-DM)* (**Figure 6A**). On *L. donovani* infection, STAT1α and BRG1 expression and occupancy, as well as H3 acetylation levels on the *CIITA* promoters are downregulated leading to decreased expression of *CIITA* and its downstream *MHC-II* genes (**Figure 6B**). Parasite infection leads to a global decrease in H3 acetylation. This further leads to downregulation of STAT1α and BRG1 resulting in decreased expression of *CIITA*. Through these series of events, the parasite can establish infection in the immunocompromised host cells. SiRNA mediated silencing of *HDAC1* leads to an increase in gene expression of *CIITA*. We believe that this knowledge will add to the development of novel prophylactic and therapeutic approaches against leishmaniasis.

## Data Availability Statement

The original contributions presented in the study are included in the article/**Supplementary Material**. Further inquiries can be directed to the corresponding authors.

## Author Contributions

Conceptualization, RMa and RMu; Methodology, RMu, RMa, HB, GR; Investigation, HB, GR, AK, and EM; Writing- original draft, HB, GR, RMa, and RMu; Writing-review and editing, HB, GR, RMa, and RMu. Funding acquisition, RMa and RMu; Supervision, RMa and RMu. All authors contributed to the article and approved the submitted version.

## Funding

RMa was funded by EMR/2016/004948 from Science and Engineering Research Board, India (https://www.serbonline.in/SERB/HomePage. do) and VI-D&P/569/2016-17/TDT/C from Department of Science and Technology, India (www.dst.gov.in). HB was supported by fellowship from CSIR, GR and EM was supported by D.S. Kothari Fellowship. The funders had no role in study design, data collection and analysis, decision to publish, or preparation of the manuscript.

## Conflict of Interest

The authors declare that the research was conducted in the absence of any commercial or financial relationships that could be construed as a potential conflict of interest.

## Publisher’s Note

All claims expressed in this article are solely those of the authors and do not necessarily represent those of their affiliated organizations, or those of the publisher, the editors and the reviewers. Any product that may be evaluated in this article, or claim that may be made by its manufacturer, is not guaranteed or endorsed by the publisher.

## References

[B1] ThermoFisher Understanding Calculations for siRNA Data: % Remaining Gene Expression and % Knockdown. Available at: https://www.thermofisher.com/in/en/home/references/ambion-tech-support/rnai-sirna/tech-notes/understanding-calculations-for-sirna-data.html (Accessed November 2019).

[B2] Bhardwaj N. Rosas L. E. Lafuse W. P. Satoskar A. R. (2005). Leishmania Inhibits STAT1-Mediated IFN-Gamma Signaling in Macrophages: Increased Tyrosine Phosphorylation of Dominant Negative STAT1beta by Leishmania Mexicana. Int. J. Parasitol 35 (1), 75–82. doi: 10.1016/j.ijpara.2004.10.018 15619518

[B3] Blanchette J. Racette N. Faure R. Siminovitch K. A. Olivier M. (1999). Leishmania-Induced Increases in Activation of Macrophage SHP-1 Tyrosine Phosphatase Are Associated With Impaired IFN-Gamma-Triggered JAK2 Activation. Eur. J. Immunol. 29 (11), 3737–3744. doi: 10.1002/(SICI)1521-4141(199911)29:11<3737::AID-IMMU3737>3.0.CO;2-S 10556830

[B4] Cheeseman K. Weitzman J. B. (2015). Host-Parasite Interactions: An Intimate Epigenetic Relationship. Cell Microbiol. 17 (8), 1121–1132. doi: 10.1111/cmi.12471 26096716

[B5] Chi T. (2004). A BAF-Centred View of the Immune System. Nat. Rev. Immunol. 4 (12), 965–977. doi: 10.1038/nri1501 15573131

[B6] Croken M. M. Nardelli S. C. Kim K. (2012). Chromatin Modifications, Epigenetics, and How Protozoan Parasites Regulate Their Lives. Trends Parasitol 28 (5), 202–213. doi: 10.1016/j.pt.2012.02.009 22480826 PMC3340475

[B7] Darnell J. E. Jr. Kerr I. M. Stark G. R. (1994). Jak-STAT Pathways and Transcriptional Activation in Response to IFNs and Other Extracellular Signaling Proteins. Science 264 (5164), 1415–1421. doi: 10.1126/science.8197455 8197455

[B8] Debrabant A. Joshi M. B. Pimenta P. F. Dwyer D. M. (2004). Generation of Leishmania Donovani Axenic Amastigotes: Their Growth and Biological Characteristics. Int. J. Parasitol 34 (2), 205–217. doi: 10.1016/j.ijpara.2003.10.011 15037106

[B9] Decker T. Lew D. J. Mirkovitch J. Darnell J. E. Jr. (1991). Cytoplasmic Activation of GAF, an IFN-Gamma-Regulated DNA-Binding Factor. EMBO J. 10 (4), 927–932. doi: 10.1002/j.1460-2075.1991.tb08026.x 1901265 PMC452736

[B10] de Ruijter A. J. van Gennip A. H. Caron H. N. Kemp S. van Kuilenburg A. B. (2003). Histone Deacetylases (HDACs): Characterization of the Classical HDAC Family. Biochem. J. 370 (Pt 3), 737–749. doi: 10.1042/BJ20021321 12429021 PMC1223209

[B11] Fernandez-Figueroa E. A. Imaz-Rosshandler I. Castillo-Fernandez J. E. Miranda-Ortiz H. Fernandez-Lopez J. C. Becker I.. (2016). Down-Regulation of TLR and JAK/STAT Pathway Genes Is Associated With Diffuse Cutaneous Leishmaniasis: A Gene Expression Analysis in NK Cells From Patients Infected With Leishmania Mexicana. PloS Negl. Trop. Dis. 10 (3), e0004570. doi: 10.1371/journal.pntd.0004570 27031998 PMC4816531

[B12] Forget G. Gregory D. J. Olivier M. (2005). Proteasome-Mediated Degradation of STAT1alpha Following Infection of Macrophages With Leishmania Donovani. J. Biol. Chem. 280 (34), 30542–30549. doi: 10.1074/jbc.M414126200 15983048

[B13] Forget G. Gregory D. J. Whitcombe L. A. Olivier M. (2006). Role of Host Protein Tyrosine Phosphatase SHP-1 in Induced Inhibition of Nitric Oxide Production. Infect. Immun. Nov; 74 (11), 6272–6279. doi: 10.1128/IAI.00853-05 PMC1695482 17057094

[B14] Forget G. Siminovitch K. A. Brochu S. Rivest S. Radzioch D. Olivier M. (2001). Role of Host Phosphotyrosine Phosphatase SHP-1 in the Development of Murine Leishmaniasis. Eur. J. Immunol. 31 (11), 3185–3196. doi: 10.1002/1521-4141(200111)31:11<3185::aid-immu3185>3.0.co;2-j 11745335

[B15] Galan J. E. Cossart P. (2005). Host-Pathogen Interactions: A Diversity of Themes, a Variety of Molecular Machines. Curr. Opin. Microbiol. 8 (1), 1–3. doi: 10.1016/j.mib.2004.12.015 15694849

[B16] Garcia-Garcia J. C. Barat N. C. Trembley S. J. Dumler J. S. (2009). Epigenetic Silencing of Host Cell Defense Genes Enhances Intracellular Survival of the Rickettsial Pathogen Anaplasma Phagocytophilum. PloS Pathog. 5 (6), e1000488. doi: 10.1371/journal.ppat.1000488 19543390 PMC2694362

[B17] Gotthardt D. Sexl V. (2016). STATs in NK-Cells: The Good, the Bad, and the Ugly. Front. Immunol. 7. doi: 10.3389/fimmu.2016.00694 PMC5241313 28149296

[B18] Goyard S. Segawa H. Gordon J. Showalter M. Duncan R. Turco S. J.. (2003). An *In Vitro* System for Developmental and Genetic Studies of Leishmania Donovani Phosphoglycans. Mol. Biochem. Parasitol 130 (1), 31–42. doi: 10.1016/s0166-6851(03)00142-7 14550894

[B19] Herwaldt B. L. (1999). Leishmaniasis. Lancet 354 (9185), 1191–1199. doi: 10.1016/S0140-6736(98)10178-2 10513726

[B20] Kouzarides T. (2007). Chromatin Modifications and Their Function. Cell 128 (4), 693–705. doi: 10.1016/j.cell.2007.02.005 17320507

[B21] Kwan W. C. McMaster W. R. Wong N. Reiner N. E. (1992). Inhibition of Expression of Major Histocompatibility Complex Class II Molecules in Macrophages Infected With Leishmania Donovani Occurs at the Level of Gene Transcription *via* a Cyclic AMP-Independent Mechanism. Infect. Immun. 60 (5), 2115–2120. doi: 10.1128/iai.60.5.2115-2120.1992 1314226 PMC257124

[B22] Lang C. Hildebrandt A. Brand F. Opitz L. Lüder C. G. K.. (2012). Impaired Chromatin Remodelling at STAT1-Regulated Promoters Leads to Global Unresponsiveness of Toxoplasma gondii-Infected Macrophages to IFN-γ. PloS Pathog. 8 (1), e1002483. doi: 10.1371/journal.ppat.1002483 22275866 PMC3262016

[B23] Lee A. J. Ashkar A. A. (2018). The Dual Nature of Type I and Type II Interferons. Front. Immunol. 9. doi: 10.3389/fimmu.2018.02061 PMC6141705 30254639

[B24] Manhas R. Anand S. Tripathi P. Madhubala R. (2014). Deletion of Vitamin C Biosynthesis Enzyme, Arabino-1, 4-Lactone Oxidase in Leishmania Donovani Results in Increased Pro-Inflammatory Responses From Host Immune Cells. Mol. Microbiol. 91 (6), 1227–1239. doi: 10.1111/mmi.12530 24456202

[B25] Marr A. K. MacIsaac J. L. Jiang R. Airo A. M. Kobor M. S. McMaster W. R. (2014). Leishmania Donovani Infection Causes Distinct Epigenetic DNA Methylation Changes in Host Macrophages. PloS Pathog. 10 (10), e1004419. doi: 10.1371/journal.ppat.1004419 25299267 PMC4192605

[B26] Martiny A. Meyer-Fernandes J. R. de Souza W. Vannier-Santos M. A. (1999). Altered Tyrosine Phosphorylation of ERK1 MAP Kinase and Other Macrophage Molecules Caused by Leishmania Amastigotes. Mol. Biochem. Parasitol 102 (1), 1–12. doi: 10.1016/s0166-6851(99)00067-5 10477171

[B27] Matte C. Descoteaux A. (2010). Leishmania Donovani Amastigotes Impair Gamma Interferon-Induced STAT1alpha Nuclear Translocation by Blocking the Interaction Between STAT1alpha and Importin-Alpha5. Infect. Immun. 78 (9), 3736–3743. doi: 10.1128/IAI.00046-10 20566692 PMC2937469

[B28] Muhlethaler-Mottet A. Otten L. A. Steimle V. Mach B. (1997). Expression of MHC Class II Molecules in Different Cellular and Functional Compartments is Controlled by Differential Usage of Multiple Promoters of the Transactivator CIITA. EMBO J. 16 (10), 2851–2860. doi: 10.1093/emboj/16.10.2851 9184229 PMC1169893

[B29] Nandan D. Lo R. Reiner N. E. (1999). Activation of Phosphotyrosine Phosphatase Activity Attenuates Mitogen-Activated Protein Kinase Signaling and Inhibits C-FOS and Nitric Oxide Synthase Expression in Macrophages Infected With Leishmania Donovani. Infect. Immun. 67 (8), 4055–4063. doi: 10.1128/IAI.67.8.4055-4063.1999 10417174 PMC96702

[B30] Nandan D. Reiner N. E. (1995). Attenuation of Gamma Interferon-Induced Tyrosine Phosphorylation in Mononuclear Phagocytes Infected With Leishmania Donovani: Selective Inhibition of Signaling Through Janus Kinases and Stat1. Infect. Immun. 63 (11), 4495–4500. doi: 10.1128/IAI.63.11.4495-4500.1995 7591091 PMC173640

[B31] Ni Z. Abou El Hassan M. Xu Z. Yu T. Bremner R. (2008). The Chromatin-Remodeling Enzyme BRG1 Coordinates CIITA Induction Through Many Interdependent Distal Enhancers. Nat. Immunol. 9 (7), 785–793. doi: 10.1038/ni.1619 18500344

[B32] Ni Z. Karaskov E. Yu T. Callaghan S. M. Der S. Park D. S.. (2005). Apical Role for BRG1 in Cytokine-Induced Promoter Assembly. Proc. Natl. Acad. Sci. U.S.A. 102 (41), 14611–14616. doi: 10.1073/pnas.0503070102 16195385 PMC1253546

[B33] Olivier M. Gregory D. J. Forget G. (2005). Subversion Mechanisms by Which Leishmania Parasites can Escape the Host Immune Response: A Signaling Point of View. Clin. Microbiol. Rev. 18 (2), 293–305. doi: 10.1128/CMR.18.2.293-305.2005 15831826 PMC1082797

[B34] Pai R. K. Askew D. Boom W. H. Harding C. V. (2002). Regulation of Class II MHC Expression in APCs: Roles of Types I, III, and IV Class II Transactivator. J. Immunol. 169 (3), 1326–1333. doi: 10.4049/jimmunol.169.3.1326 12133955

[B35] Patne K. Rakesh R. Arya V. Chanana U. B. Sethy R. Swer P. B.. (2017). BRG1 and SMARCAL1 Transcriptionally Co-Regulate DROSHA, DGCR8 and DICER in Response to Doxorubicin-Induced DNA Damage. Biochim. Biophys. Acta Gene Regul. Mech. 1860 (9), 936–951. doi: 10.1016/j.bbagrm.2017.07.003 28716689

[B36] Pattenden S. G. Klose R. Karaskov E. Bremner R. (2002). Interferon-Gamma-Induced Chromatin Remodeling at the CIITA Locus Is BRG1 Dependent. EMBO J. 21 (8), 1978–1986. doi: 10.1093/emboj/21.8.1978 11953317 PMC125964

[B38] Proudfoot L. Nikolaev A. V. Feng G. J. Wei W. Q. Ferguson M. A. Brimacombe J. S.. (1996). Regulation of the Expression of Nitric Oxide Synthase and Leishmanicidal Activity by Glycoconjugates of Leishmania Lipophosphoglycan in Murine Macrophages. Proc. Natl. Acad. Sci. U.S.A. 93 (20), 10984–10989. doi: 10.1073/pnas.93.20.10984 8855295 PMC38270

[B39] Ramsauer K. Farlik M. Zupkovitz G. Seiser C. Kroger A. Hauser H.. (2007). Distinct Modes of Action Applied by Transcription Factors STAT1 and IRF1 to Initiate Transcription of the IFN-Gamma-Inducible Gbp2 Gene. Proc. Natl. Acad. Sci. U.S.A. 104 (8), 2849–2854. doi: 10.1073/pnas.0610944104 17293456 PMC1815270

[B40] Ray M. Gam A. A. Boykins R. A. Kenney R. T. (2000). Inhibition of Interferon-Gamma Signaling by Leishmania Donovani. J. Infect. Dis. 181 (3), 1121–1128. doi: 10.1086/315330 10720539

[B41] Roy G. Brar H. K. Muthuswami R. Madhubala R. (2020). Epigenetic Regulation of Defense Genes by Histone Deacetylase1 in Human Cell Line-Derived Macrophages Promotes Intracellular Survival of Leishmania Donovani. PloS Negl. Trop. Dis. 14 (4), e0008167. doi: 10.1371/journal.pntd.0008167 32275661 PMC7176143

[B42] Shen W. Xu C. Huang W. Zhang J. Carlson J. E. Tu X.. (2007). Solution Structure of Human Brg1 Bromodomain and its Specific Binding to Acetylated Histone Tails. Biochemistry 46 (8), 2100–2110. doi: 10.1021/bi0611208 17274598

[B43] Singh A. K. Pandey R. K. Siqueira-Neto J. L. Kwon Y. J. Freitas-Junior L. H. Shaha C.. (2015). Proteomic-Based Approach to Gain Insight Into Reprogramming of THP-1 Cells Exposed to Leishmania Donovani Over an Early Temporal Window. Infect. Immun. 83 (5), 1853–1868. doi: 10.1128/IAI.02833-14 25690103 PMC4399049

[B44] Singh B. Singh O. P. Singh N. Singh S. S. Sundar S. (2019). Abnormal B-Cell Subset and Blimp-1-Mediated Humoral Responses Associated With Visceral Leishmaniasis Pathogenesis. Am. J. Trop. Med. Hyg 100 (4), 816–821. doi: 10.4269/ajtmh.18-0350 30793688 PMC6447117

[B45] Steimle V. Siegrist C. A. Mottet A. Lisowska-Grospierre B. Mach B. (1994). Regulation of MHC Class II Expression by Interferon-Gamma Mediated by the Transactivator Gene CIITA. Science 265 (5168), 106–109. doi: 10.1126/science.8016643 8016643

[B46] Trotter K. W. Archer T. K. (2008). The BRG1 Transcriptional Coregulator. Nucl. Recept Signal 6, e004. doi: 10.1621/nrs.06004 18301784 PMC2254329

[B47] Wright K. L. Ting J. P. (2006). Epigenetic Regulation of MHC-II and CIITA Genes. Trends Immunol. 27 (9), 405–412. doi: 10.1016/j.it.2006.07.007 16870508

[B48] Zika E. Greer S. F. Zhu X. S. Ting J. P. (2003). Histone Deacetylase 1/Msin3a Disrupts Gamma Interferon-Induced CIITA Function and Major Histocompatibility Complex Class II Enhanceosome Formation. Mol. Cell Biol. 23 (9), 3091–3102. doi: 10.1128/mcb.23.9.3091-3102.2003 12697811 PMC153210

[B49] Zupkovitz G. Tischler J. Posch M. Sadzak I. Ramsauer K. Egger G.. (2006). Negative and Positive Regulation of Gene Expression by Mouse Histone Deacetylase 1. Mol. Cell Biol. 26 (21), 7913–7928. doi: 10.1128/MCB.01220-06 16940178 PMC1636735

