# Supplementary material for: Chromatin-Remodeling Factor BRG1 Is a Negative Modulator of L. donovani in IFNγ Stimulated and Infected THP-1 Cells
Source: Front Cell Infect Microbiol. 2022 Apr 1;12:860058. doi: 10.3389/fcimb.2022.860058 (PMC9011159; doi:10.3389/fcimb.2022.860058)
Supplement: Supplementary file 1 [file DataSheet_1.docx]

|  | Gene name | Primer sequence (5’-3’) | |
| --- | --- | --- | --- |
| RT-PCR genes | *BRG1* | BRG1 F  BRG1 R | GAGCAACCAACCACAAAGTG  GCTGAGCAGGAAGATGAAGTA |
|  | *STAT1α* | STAT1 F  STAT1 R | CCAGGCTCTTGATTTCATGC  AATTCTGGAAAACGCCCAG |
|  | *CIITA* | CIITA F  CIITA R | GACAGGAAGCAGAAGGTGCT  GCCCAGTACATGTGCATCAG |
|  | *HLA-DR* | HLA-DR F  HLA-DR R | AGTCCCTGTGCTAGGATTTTTCA  ACATAAACTCGCCTGATTGGTC |
|  | *HLA-DM* | HLA-DM F  HLA-DM R | GTCCAGAGGGTTTCCTATCGC  CACAGGGACGGAATGATGCT |
|  | *HDAC1* | HDAC1 F  HDAC1 R | GACGGGGATGTTGGAAATTA  CATCTCCTCAGCATTGGCTT |
| Housekeeping Gene | *RNU6A (*RNA, U6 Small Nuclear 1) | RNU 6A-F  RNU 6A-R | GGCCCAGCAGTACCTGTTTA  AGATGGCGGAGGTGCAG |
| ChIP genes | *CIITA* (promoter I) | CIITA pI F  CIITA pI R | GACCTATTACCCTATAGGTCCT  TGGGGTTCCTGATGAACG |
|  | *CIITA* (promoter IV) | CIITA pIV F  CIITA pIV R | GCAGTTGGGATGCCACTTCT  TCT CCC TCC CGC CAG CT |
|  | *TOP1* (promoter) | TOP1 F  TOP1 R | CCTTATGCAAATCACAGCGGAG  CGCTTTTAAACAACACGTCGG |

**Table S1.** Genes and primers used in the above study.

**Fig. S1: Effect of *L. donovani* infection on STAT1α IFNγ stimulated host cells.** THP-1 cells were differentiated by PMA for 48 h, then infected with *L. donovani* for 3 h followed by stimulation with IFNγ or were left unstimulated. **(A)** Cells were harvested at 0, 3, 6 and 24 h post-stimulation and total RNA was extracted from different samples for quantifying *STAT1α* expression by qPCR. *RNU6A* was used as a housekeeping gene. The mRNA levels were expressed using the 2^-ΔΔCT^ method. Transcript levels of 0 h samples were used for data normalization. Pink color represents: - IFNγ - *L. donovani*; green color represents: - IFNγ + *L. donovani*; blue represents: + IFNγ - *L. donovani*; orange color represents: + IFNγ + *L. donovani.* **(B)** Cells were harvested at 0 and 6 h post-stimulation for detecting STAT1α protein expression by immunoblotting using an anti-STAT1α antibody. Protein levels were quantitated by densitometry using ImageJ software. STAT1α protein levels were normalized with the respective β-actin protein levels. **(C)** Translocation of STAT1α into the nucleus was observed by immunofluorescence microscopy. Cells were fixed 6 h post-stimulation with methanol and incubated with the anti-STAT1α antibody. (I) represents cells under DIC. (II) represents THP-1 and parasite nuclei stained with DAPI. (III) represents cells stained with the anti-STAT1α antibody. (IV) represents the merged image and (V) zoomed image. **(D)** To detect the translocation of STAT1α in the host cell nuclei under different conditions, anti-STAT1α fluorescence intensity was calculated for each cell and plotted. Pink color represents: - IFNγ - *L. donovani*; green color represents: - IFNγ + *L. donovani*; blue represents: + IFNγ - *L. donovani*; orange color represents: + IFNγ + *L. donovani.*

**(D)**


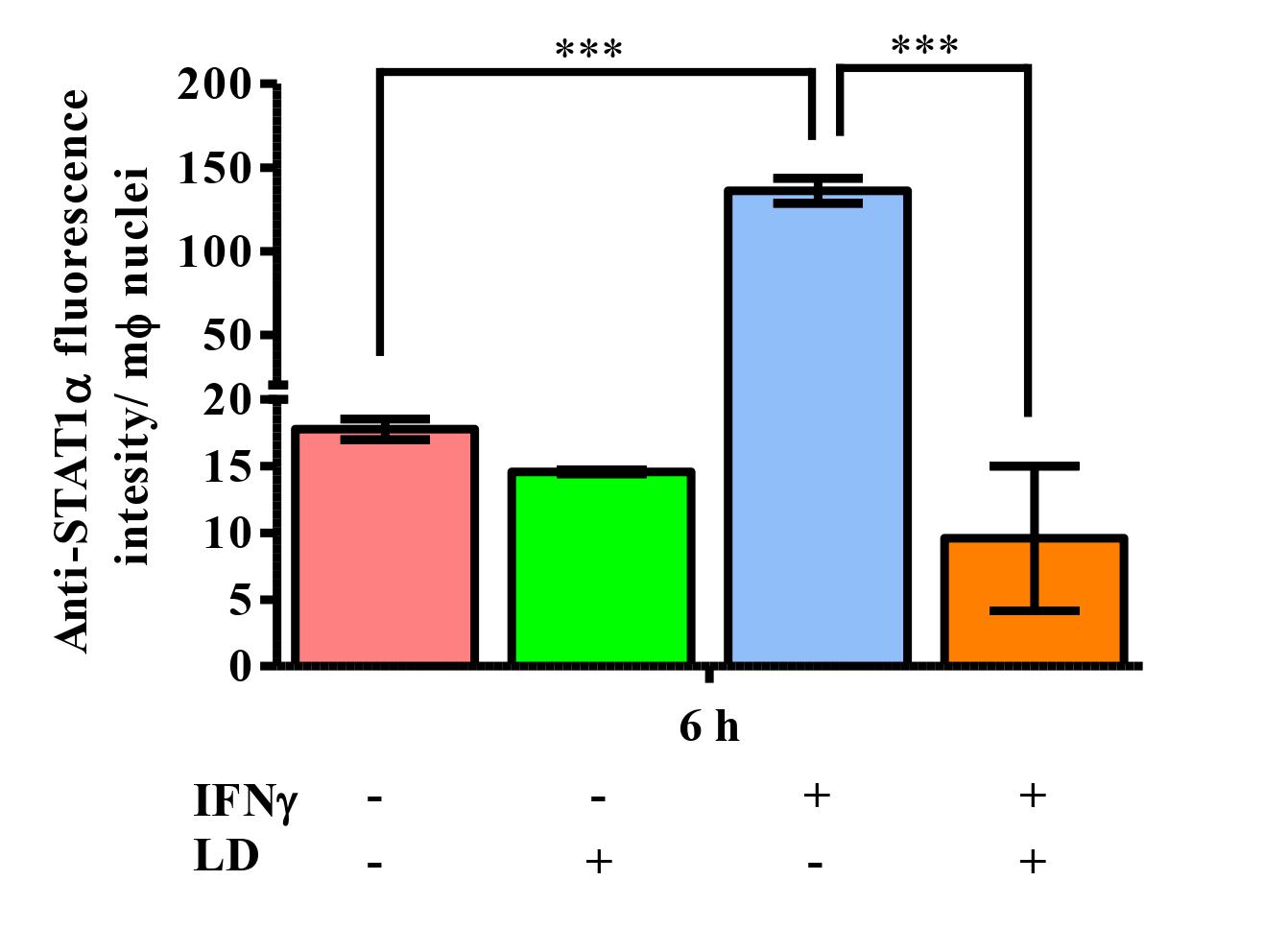


**Fig. S2:** ***L. donovani* impairs expression of host *CIITA* and *MHC-II* genes*.*** THP-1 cells (10^6^ cells/ml) were either infected or not with *L. donovani* at an MOI of 20:1(parasite: macrophage) followed by either IFNγ stimulation for 30 min or left unstimulated. Cells were harvested at 0, 6 and 24 h post-stimulation and mRNA expression of **(A)** *CIITA* and **(B)** *HLA-DR* and **(C)** *HLA-DM* was analysed. Total RNA was isolated and examined by qPCR. *RNU6A* was used as a housekeeping gene. Data analysis was done using the 2^-ΔΔCT^ method. For data normalization and calculating relative abundance, transcript levels of respective genes at 0 h were used. Pink color represents: - IFNγ - *L. donovani*; green color represents: - IFNγ + *L. donovani*; blue represents: + IFNγ - *L. donovani*; orange color represents: + IFNγ + *L. donovani.*

**Fig. S3: Impact of IFNγ on parasite load within the macrophages.** THP-1 cells were treated with 50 ng/ml of PMA for 48 h. They were infected with promastigotes at an MOI of 20:1 for 3 h followed by stimulation with IFNγ for 30 min. The cells were afterwards sustained in RPMI1640 for 6 h and 24 h. For counting of intracellular parasite load, Giemsa staining was performed at 6 h and 24 h post stimulation. Green color represents: - IFNγ + *L. donovani*; orange color represents: + IFNγ + *L. donovani.*


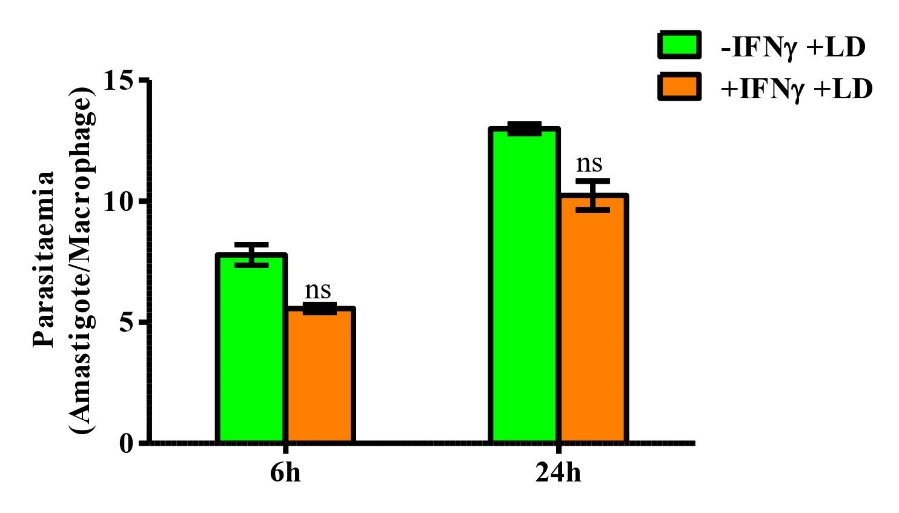


**Fig. S4: Effect of heat-killed leishmania (HKLD) on the expression of BRG1 and STAT1α with the stimulation of IFNγ.** *Leishmania* promastigotes were incubated at 65^0^C for 45 min to generated heat-killed *L. donovani* (HKLD) (Marr et al., 2014). THP-1 cells were treated with 50 ng/ml of PMA for 48 h and then infected with HKLD at a MOI of 20:1 for 3 h followed by stimulation with IFNγ for 30 min, similar to infection with live parasites. The cells were harvested at 3, 6 and 24 h post stimulation. Total RNA was extracted and *BRG1* and *STAT1α* expression were quantified by qPCR. *RNU6A* was used as a housekeeping gene. The mRNA levels were expressed using the 2^-ΔΔCT^ method. Transcript levels of 0 h samples were used for data normalization. Green color represents: - IFNγ + HKLD; orange color represents: + IFNγ + HKLD*.*

**(A)**

**(B)**

**Fig. S5: Occupancy of STAT1α and BRG1 at promoter region of *TOP1*.** To study the occupancy of STAT1α and BRG1 at *TOP1* promoter, a negative control region, ChIP assay was used. Chromatin from stimulated and/or infected cells was pulled down by STAT1α or BRG1 antibody, followed by qPCR using primers specific for *TOP1* promoter region. No antibody and 0 h chromatin extract were used as controls. Pink color represents: - IFNγ - *L. donovani*; green color represents: - IFNγ + *L. donovani*; blue represents: + IFNγ - *L. donovani*; orange color represents: + IFNγ + *L. donovani.*

**Fig. S6: Effect of *BRG1* overexpressor on parasite survival in THP-1 cells.** Differentiated THP-1 cells were infected with *L. donovani* for 3 h*,* following which they were either stimulated with IFNγ or not. The cells were then transiently transfected for 48 h with either 1.5 μg of plasmid overexpressing *BRG1* (OE) or control. Giemsa staining was done to study the survivability of parasites visually. Green color represents: - IFNγ + *L. donovani*; orange color represents: + IFNγ + *L. donovani.*

**(A)**
